# Haplotype GWAS in Colorectal Cancer Patients with a Family History of Gastric or Prostate Cancer

**DOI:** 10.3390/ijms27010547

**Published:** 2026-01-05

**Authors:** David Kudrén, Linda Waage, Johanna Samola Winnberg, Mats Lindblad, Chunde Li, Annika Lindblom, Litika Vermani

**Affiliations:** 1 Department of Oncology, Södersjukhuset, 11833 Stockholm, Sweden; chunde.li@ki.se; 2 Department of Clinical Science and Education, Karolinska Institutet, 11833 Stockholm, Sweden; linda.waage@ki.se; 3 Department of Urology, Södersjukhuset, 11833 Stockholm, Sweden; 4 Division of Surgery, Department of Clinical Science Intervention and Technology (CLINTEC), Karolinska Institutet, 17177 Stockholm, Sweden; johanna.samola.winnberg@ki.se (J.S.W.); mats.lindblad@ki.se (M.L.); 5 Department of Upper Abdominal Diseases, Karolinska University Hospital, 17166 Stockholm, Sweden; 6 Department of Molecular Medicine and Surgery, Karolinska Institutet, 17177 Stockholm, Sweden; litikavermani@cacharcancerhospital.org; 7 Department of Clinical Genetics, Karolinska University Hospital, 17166 Stockholm, Sweden; 8 Dr. S Krishnamurthi Centre for Research and Education in Cancer, Cachar Cancer Hospital and Research Centre, Silchar 788015, India

**Keywords:** haplotype, GWAS, colorectal cancer, gastric cancer, prostate cancer, familial, genetic, CRC syndrome, predisposition, cancer risk

## Abstract

Previous haplotype Genome Wide Association Studies (GWASs) have suggested several rare loci with a shared increased risk of colorectal, gastric, and prostate cancer. This study aimed to find out more about markers specifically addressing the shared risk of colorectal and gastric cancer, as well as the shared risk of colorectal and prostate cancer. One analysis used 426 colorectal cancer cases with gastric cancer, with no prostate cancer cases in their families, and another analysis used 324 colorectal cancer cases with prostate cancer but no gastric cancer among relatives. The computational program PLINK v1.07 was used for the analysis and for the calculation of corresponding ORs, standard errors, and 95% confidence intervals (CI). The study found support for the loci from previous studies and many new loci with a shared risk of colorectal cancer and gastric cancer. There were no significant loci from the second analysis for a shared risk of colorectal and prostate cancer. Altogether, more than 100 new loci with a shared risk of colorectal cancer and gastric cancer were suggested. A shared risk of colorectal and prostate cancer at some loci could not be ruled out. Haplotype GWAS has again demonstrated its ability to find rare risk loci mostly associated with coding genes.

## 1. Introduction

Colorectal cancer (CRC) sometimes occurs in a familial setting, and today several genes are known to be associated with an increased risk of CRC [1]. The high-risk genes have also been considered causing genetic syndromes, with an increased risk of not only CRC but also many other cancers, such as in Lynch Syndrome or Familial Adenomatous Polyposis Coli (FAP) syndromes, caused by mutations in the DNA mismatch repair genes (*MLH1*, *MSH2*, *MSH6*, and *PMS2*) and the *APC* or *MUTYH* genes, respectively [1]. Less frequent mutations occur in genes such as *SMAD4*, *BMPRIA*, *STK11*, and *PTEN*, which are also known to cause CRC and other cancers in the context of cancer syndromes [1]. However, the known high-risk syndromes do not explain all familial CRC, and other syndromes are possible, conceivably with a relatively moderate increased risk [2].

Common diseases are suggested to be caused by an interplay between predisposing genetic variants in low-risk genes and environmental factors, as in the concept of complex disease [3]. To date, Genome Wide Association Studies (GWASs) have therefore been used to identify common genetic loci associated with an increased risk of CRC, as well as other common disorders [4,5,6,7]. Many GWASs on CRC have been published and have identified more than 200 independent risk associations [8,9]. Studies examining pleiotropy across multiple cancer-associated loci have searched for shared genetic risk loci for common mechanisms of cancer development and progression in malignancy [10]. If the increased risk is modest, these genes are still associated with multiple tumors and could constitute low-risk syndromes.

**Figure 1:**
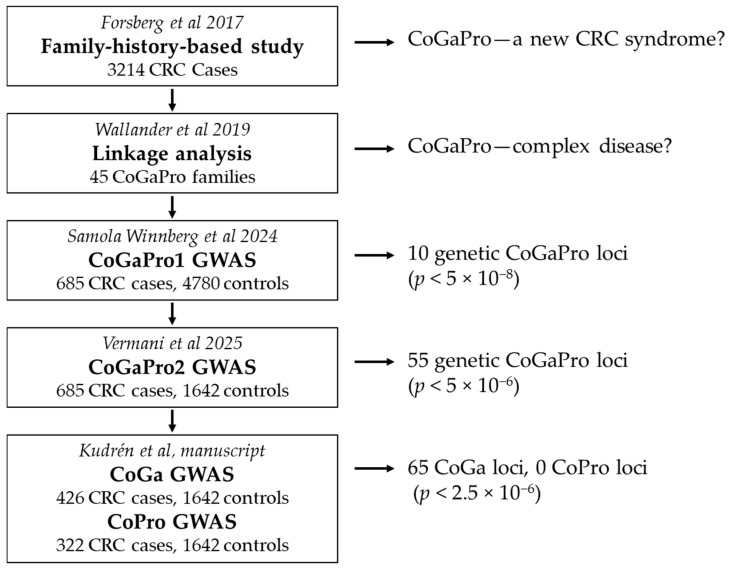
A study of family history across 3214 CRC patients suggested a putative new syndrome where the subject had an increased risk of CRC and other cancers, primarily gastric and prostate cancer [11]. Next, linkage analysis, used in 45 families who possibly have this putative syndrome, was undertaken without any suggested loci, and therefore, a complex rather than monogenic disease was suggested [12]. Therefore, a series of GWASs was undertaken to find out if the selection of CRC cases with a family history of gastric and/or prostate cancer suggested several loci [13,14]. Finally, in the current study, CRC cases with either gastric or prostate cancer were used for the study.

We have published a series of studies on a hypothetical CRC syndrome with a shared risk of colorectal, gastric, and prostate cancer (Figure 1). A first study suggested familial colorectal cancer associated with a possible increased risk for other tumors, primarily gastric and prostate cancer [11]. Linkage analysis in families with colorectal, gastric, and/or prostate cancer was undertaken to study the pattern of inheritance in the underlying hypothetical syndrome. This analysis did not suggest any high-penetrant disease-associated locus, and instead, a complex disease for this putative low-risk syndrome was suggested [12]. To test this hypothesis, first, a haplotype genome-wide association study (GWAS) was conducted involving CRC patients who had gastric and/or prostate cancer among their relatives (CoGaPro1) [13]. That study used healthy, unrelated Swedish twins as controls. The result gave support for 10 risk loci, all with known coding cancer genes, associated with an increased risk of colorectal and possibly also gastric and prostate cancer [13]. Since another cohort of controls, better matched geographically, and genotyped with the same chip, was available, it was possible to perform a second haplotype GWAS (CoGaPro2) using the same cases and new controls [14]. The ten loci from the first study were confirmed, and another 50 (ORs 1.4–6.5) were suggested, many with known coding cancer genes. The study first suggested that the association with multiple tumors could not define whether the increased risk beyond CRC concerned one or several CRC syndromes [11]. Since the current hypothesis is a complex disease with numerous risk loci, it is possible that some loci confer a shared risk for some tumors, while other loci mostly confirm a shared risk for other tumors. Thus, each low-risk syndrome locus could, in fact, constitute its own syndrome related to the specific mechanisms acting at that precise locus. Thus, to find out more about the suggested low-risk syndrome of shared risk loci, a third study was designed to again use haplotype GWAS and subsets of the same CRC cohort as before (Figure 1). However, this time, CRC cases with gastric cancer and no prostate cancer cases in their families were used in one analysis, and CRC cases with prostate cancer but no gastric cancer in their families were used in a second analysis. Both analyses used the same controls as in CoGaPro2. The aim was to find out if the results could give more information about shared risk loci and genes among these three cancer types. The resulting risk loci were to be compared to the results from our previous haplotype studies in CRC.

## 2. Results

**Figure 2:**
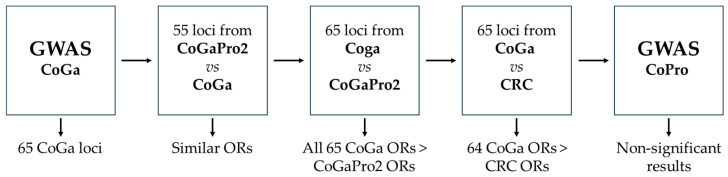
A haplotype GWAS using 426 CRC cases with gastric cancer (and no prostate cancer) in their families resulted in 65 significant loci. A previous study, CoGaPro2, had suggested 55 loci, and those 55 had similar ORs in the current study. The 65 loci from the current study were also compared to the same loci in the CoGaPro2 study, and all 65 loci had improved ORs in the current study. The 65 loci were also compared to the same loci in the first haplotype GWAS using all unselected CRC cases and all CRC cases, but one locus was improved in the current study of selected cases. The haplotype GWAS on 322 CRC cases with prostate (but no gastric) cancer in their families did not result in any significant loci.

A sliding window (1–25) haplotype GWAS was conducted using 426 CRC cases with gastric cancer (and no prostate cancer) in the relatives and 1642 healthy controls (Supplementary Tables S1–S23). After this GWAS, the results were compared to previous GWASs for more information on the risks (ORs) in this and in the previous studies (Figure 2). All haplotype results are presented in Supplementary Tables with the following columns: NSNP, number of SNPs; NHAP, number of haplotypes in the window; BP1, first base pair in haplotype; BP1, last base pair in haplotype; SNP1, first SNP in haplotype; SNP2, last SNP in haplotype; F, frequency; OR, odds ratio; Stat, squared T statistics in Wald; and P, *p*-value.

### 2.1. GWAS CoGa (Figure 2)

A total of 65 haplotypes in 62 loci had significant *p*-values, defined as *p* < 2.5 × 10^−6^, with ORs from 1.48 to 7.39 (Table 1, Supplementary Table S24). Out of these 65 haplotypes, 46 had 1 to 6 coding genes, and many had non-coding genes, RNA genes, or pseudogenes, between the first and last positions.

Two loci, on chromosome 2 (*DOCK10*) and chromosome 9 (*FCN2*, *FCN1*), suggested in CoGaPro1 (Supplementary Table S25), and twelve haplotypes at eleven loci, suggested in CoGaPro2 (Supplementary Table S26), were the same in CoGa (indicated with * in Table 1). Ten of these eleven loci involved the following genes: *CDK15*, *CROCC2*-*SNED1*, *TLR2*-*RNF175*-*SFRP2*, *WWC2*, *RELN*, *ZMYM5*-*ZMYM2*, *KLF12*, *SKAP1*, *CABLES2*, and *MIR630*.

### 2.2. CoGaPro2 vs. CoGa (Figure 2)

Since the analysis in the CoGaPro2 study and the CoGa analysis used the same micro-chip for genotyping, the same controls, and partly the same cases (those with prostate cancer had been removed), it was possible to compare all specific haplotypes suggested in CoGaPro2 to find out how the new selection of cases influenced the result in the CoGa study. The 55 haplotypes in the CoGaPro2 study were now compared to the same haplotypes in the CoGa study (Supplementary Table S27). ORs were similar in the two studies, ranging from 1.34 to 5.98 in the CoGaPro2 and from 1.35 to 6.52 in the CoGa analysis. ORs were higher in CoGaPro2 compared to CoGa at 32 haplotypes and lower in CoGaPro2 at 22 haplotypes (Supplementary Table S27). Haplotypes at four loci showed ORs in the CoGaPro2 study that were at least twice as high as the same loci in the CoGa study (Supplementary Table S27).

### 2.3. CoGa vs. CoGaPro2 (Figure 2)

The CoGa results were expected to present loci with higher OR at loci associated with CRC and gastric cancer. Thus, all haplotypes in CoGa were now compared to the same haplotypes in CoGaPro2 (Supplementary Table S28). Indeed, all 65 haplotypes in CoGa demonstrated higher ORs (range 1.48–7.39), and most with lower *p*-values compared to the same haplotypes in the CoGaPro2 study (range 1.19–4.47) (Supplementary Table S28).

### 2.4. CoGa vs. CRC (Figure 2)

Since the ORs relate to an increased risk of CRC, and we had used the full cohort of 2663 CRC cases, and the same controls in a previous study [15], we also compared the results in CoGa to those of the CRC study. All 65 haplotypes could be identified, sometimes with a slightly different length (Supplementary Table S29). For all but one locus on chromosome 9 (*FGD3*, *SUSD3*), haplotype ORs were higher (range 1.48–7.39) in the CoGa Study, compared to in the CRC Study (range 1.18–2.65) (Supplementary Table S29). Five haplotypes were statistically significant also in the CRC GWAS, two on chromosome 2, *CDK15*, and one with no gene on 2q36.1, and a further one locus on chromosome 20 (*LAMA5*) with three haplotypes (Supplementary Table S29).

### 2.5. GWAS CoPro (Figure 2)

Finally, another sliding window haplotype GWAS was conducted using 324 CRC cases with prostate cancer, but no gastric cancer in their families, and 1642 healthy controls. No statistically significant results were obtained in this analysis (Supplementary Tables S30–S52).

## 3. Discussion

This study is the third GWAS published on hypothetical cancer syndromes associated with CRC, gastric, and/or prostate cancer. The three studies have all investigated what types of familial cancers carry a high risk of developing CRC. The first two studies suggested loci where a family history of both gastric and prostate cancer was associated with a risk of CRC. The two loci (*DOCK10* and *FCN1-FCN2*) replicated from CoGaPro1, and the 11 loci replicated from CoGaPro2 (*CDK15*, *CROCC2-SNED1*, *TLR2-RNF175-SFRP2*, *WWC2*, *RELN*, *ZMYM5-ZMYM2*, *KLF12*, *SKAP1*, *CABLES2*, and *MIR630*) gave further support for these genes being associated with a risk of CRC and gastric cancer. Many of the genes related to colorectal cancer, and even gastric cancer, have been published. One study identified cfDNA methylation biomarkers detectable in blood, reflecting tumor-derived signals from gastric cancer, such as *DOCK10* [16]. *CDK15* was shown to be involved in CRC progression in the β-catenin signaling pathway [17]. The *SFRP2* gene has been published several times in relation to both CRC and gastric cancer [18]. Studies have suggested the *RELN* pathway to be involved in gastric as well as colorectal carcinogenesis [19,20]. Krüppel-like factor 12 (*KLF12*) is a transcription factor that plays a role in normal kidney development, and *KLF12* is frequently elevated in esophageal adenocarcinoma and has been reported to promote gastric cancer progression and also to be involved in colorectal cancer [21]. *SKAP1* promotes cell proliferation and invasion and is associated with poor prognosis in colorectal cancer. The gene has also been suggested to represent a biomarker and therapeutic target in gastric cancer to regulate cellular functions through *JAK1/PI3K/AKT* signaling [22].

Although only 13 loci were replicated in this third study, it does not exclude any of the loci suggested in the previous studies, since in the comparison of the loci in CoGaPro2 and CoGa, ORs were similar for all haplotypes, except at four loci, where the ORs were much lower in the CoGa study. Those loci were *CNTNAP2*, one locus without a gene on chromosome 6, *CDH13*, and *ASIC2*. The fact that ORs became much lower at these loci when CRC cases with a family history of prostate cancer were removed suggested that those four loci/genes were candidates for a risk of prostate cancer as well and needed to be investigated in further studies. The *CNTNAP2* gene was identified as a fragile site, and although fragile sites are not traditional mutational targets in cancer, they do exhibit loss of expression in multiple tumor types, suggesting that they may also function as tumor suppressors [23]. *CDH13* encodes T-cadherin, which belongs to the cadherin superfamily, which has a wide array of biological functions [24]. *CDH13* is highly conserved among species, indicating evolutionary importance [25]. Under oxidative stress, the overexpression of T-cadherin protects against endothelial cell apoptosis [26]. While T-cadherin promotes angiogenesis, loss of function is associated with several cancers, including prostate and colorectal cancer [24]. Germline mutations in *CDH1*, coding for the related protein E-cadherin, are associated with a well-described cancer syndrome, including hereditary diffuse gastric cancer [27]. *ASIC2* encodes a protein with the same name, which belongs to a family of acid-sensitive ion channels (ASICs). There are four *ASIC* genes and seven proteins. The majority of cancer research has been on *ASIC1*, whereas the roles of *ASIC2* and *ASIC3* have been explored in only a limited number of studies, but they seem related to acidification of the tumor microenvironment [28,29].

All 65 haplotypes in the CoGa study demonstrated higher ORs compared to the same haplotypes in the CoGaPro2 study, as expected. The fact that ORs became higher after removing cases with a family history of prostate cancer supported the association of a risk of CRC, as well as gastric cancer. Interestingly, six loci/haplotypes had ORs at least twice as high as those in the CoGaPro2 study. These were on chromosome 1 (*TRIT1-MYCL-MFSD2A-CAP1*), also on chromosome 1 (*KIAA0040-TNR*), on chromosome 12 (*CCND2*), chromosome 17 (*RAP1GAP2*), chromosome 18 (TGIF1), and on chromosome 22 (*SCUBE1*). It was recently reported that several tagging SNPs and haplotypes in *TRIT1* were significantly associated with a risk and clinicopathological features of gastric cancer in a Chinese population [30]. The other three genes in that haplotype, *MYCL*, *MFSD2A*, and *CAP1,* have also been suggested in gastric carcinogenesis [31,32,33]. *CCND2* expression was suggested to be an independent prognostic factor for overall survival in patients with gastric cancer [34]. *TGIF1*, a transcriptional corepressor involved in breast and lung cancer, has also been suggested to promote colorectal cancer through activating Wnt/β-catenin signaling [35]. *SCUBE1* is a novel mammalian *EGF*-related protein that has been suggested as a potential new biomarker for gastric cancer [36].

The 65 haplotypes from the CoGa study were also compared to those from a previous GWAS of all CRC cases. The fact that the ORs for all but one locus were higher in the CoGa study further supported the hypothesis that the selection of cases based on a family history of gastric cancer was relevant and that there are loci associated with a risk of both CRC and gastric cancer.

The haplotype GWAS conducted on 324 CRC cases with prostate cancer in their families and 1642 healthy controls did not result in any statistically significant results. However, since prostate cancer is common in the population, the selection of CRC cases based on a family history of at least one case of prostate cancer in the family probably was not strict enough to result in a relevant selection of cases. Thus, it was more difficult to obtain significant results. Still, many genes were suggested in this GWAS, even with non-significant *p*-values. Some of them were already published in relation to prostate cancer (Supplementary Table S53). *PUM1* encodes the RNA-binding protein Pumilio-1, which is highly conserved. It is highly expressed in many cancers, including prostate cancer, and is correlated with reduced survival. *PUM1* mediates translational repression of *CDKN1B* [37]. *KCNN3*, which encodes a potassium calcium-activated channel, is a tumor-suppressor gene that has been observed to be downregulated in prostate cancer [38,39]. *CCT3* encodes one of eight subunits of chaperons, which catalyze the folding of proteins in cell division, proliferation, and apoptosis. Increased expression is associated with prostate cancer and other cancers [40]. *LEF1* regulates the expression of the androgen receptor and is associated with the invasive ability of prostate cancer [41]. *RREB1* promotes prostate cancer by activating the transcription of *SNHG4*, which promotes DNA damage repair, the cell cycle, and resistance to the androgen-receptor antagonist enzalutamide [42]. *CSMD1* is one of several genes that are typically overexpressed in monocytes in prostate cancer and has been suggested as a biomarker differentiating prostate cancer from benign prostatic hyperplasia [43]. However, the gene has also been suggested as a tumor suppressor in other cancers [44,45]. *DLC1* regulates E-cadherin and suppresses highly metastatic prostate cancer cell invasion by modulating the Rho pathway. Overexpression of *DLC1* markedly suppresses proliferation and cell cycle progression [46,47]. *TNFRSF10C* is one of the most frequently deleted loci in prostate cancer and other cancers as well. In prostate cancer, very often the gene is either hemizygously deleted or has its promoter CGI hypermethylated [48]. The gene is a receptor inducing tumor apoptosis in multiple malignancies [49,50]. *NRG1* encodes the protein neuroligin-1. Protein levels are elevated, both in serum and in tumor tissue, in castration-resistant prostate cancer. High neuroligin levels also correlate with high levels of prostate-specific antigen (PSA) and Gleason grading, both associated with more aggressive disease, suggesting that *NRG1* could be a marker for predicting progression of prostate cancer [51]. *TMEM64* downregulates the expression of Wnt3a, leading to less activation of the β-catenin-dependent signaling pathway. That pathway, and Wnt signaling otherwise, affects the tumor microenvironment and promotes therapy resistance [52]. *DEPTOR* is a tumor suppressor gene. The protein binds to mTORC1 and mTORC2 complexes and blocks their activities, and thereby suppresses protein synthesis, cell growth, proliferation, and survival [53]. *SAMHD1* has recently been identified as associated with prostate cancer, with rare mutations carrying a very high risk [54,55].

Overall, the first two studies on this hypothetical syndrome gave support for several loci with a shared risk for CRC, gastric, and prostate cancer. The present study, focusing only on CRC cases with either gastric or prostate cancer in their family members, found new loci with a shared risk for CRC and gastric cancer, and even, possibly, prostate cancer. In the end, it was suggested that the risk of CRC, gastric, and prostate cancer differs at different loci. An obvious limitation of this and the previous studies is that only CRC cases were available for study. A putative risk of gastric and/or prostate cancer and even other tumor types in CRC patients, as well as their relatives, motivates further studies. The family histories of all CRC cases in all studies include many more tumors, and in total, the number of tumors reported in this study was 505 gastric, 139 breast, 69 gynecological, 71 lung, 56 bladder, 56 leukemia/lymphoma, 36 CNS, 22 kidney, 21 pancreatic, 10 gall bladder, and 330 unspecified tumors. The absolute risk at these loci is of interest. Most of the published GWAS CRC loci are common and mostly with ORs below two. However, at most loci suggested in our haplotype studies, rare loci and higher ORs were found. The first study had ORs > 3, the second had ORs > 6, and, in this third study, ORs > 7 were seen. It will be important to replicate these results to determine the risk at some of these loci to find out the risk for CRC, gastric, and prostate, as well as other cancers. Thus, these results need to be confirmed in larger studies, and in other populations, including patients with other cancer types for study.

## 4. Materials and Methods

### 4.1. Cases and Controls for GWAS

Cases were selected as a part of a multi-center study, the Colorectal Cancer Low-risk study, with newly diagnosed CRC patients from the middle of Sweden between 2004 and 2009 [11]. Detailed information regarding cancer occurrences in the family, comprising first- and second-degree relatives and cousins, was recorded. Based on family history and the pathology and molecular testing for microsatellite instability (MSI), known cases of FAP and Lynch syndrome were excluded from the study. The selection criterion for the patients to be included in this study was having at least one case of gastric or prostate cancer among close relatives. In total, 426 cases fulfilling this criterion were genotyped and included as cases in one analysis. The relatives of these CRC cases had various cancers in different locations, with gastric cancer as the most common malignancy, and with other various types of cancer except prostate cancer. One other GWAS analysis used 324 CRC cases with at least one case of prostate cancer and no gastric cancer cases among the relatives. In total, 1642 controls from the low-risk study were used for the analysis (536 spouses of the cases, and 1106 healthy blood donors from the same geographical region). The demographics of cases are shown in Table 2. No information was obtained for control persons.

### 4.2. Genotyping, Quality Control, and Haplotype GWAS

DNA was extracted from blood using standard procedures in the lab. The genotyping for both cases and controls was performed at the Centre for Inherited Disease Research at Johns Hopkins University, US, using the Illumina Infinium^®^ OncoArray-500K, Illumina, Inc., San Diego, CA, USA [56]. The first QC was performed within the CORECT study [57], and a second QC was performed at Karolinska Institutet [15]. For the haplotype GWAS analysis, to examine the association between one single SNP, or a haplotype, and cancer risk, a logistic regression model was employed using a sliding window approach. The computational program PLINK v1.07 was used for the analysis and for the calculation of corresponding OR, standard errors, and 95% confidence intervals (CI) [58]. The following parameters were applied while using PLINK v1.07: “hap-logistic” (haplotype logistic regression analysis), “hap-window 1–25” (sliding window sizes 1 to 25), and default settings, which included haplotypes phasing with the E-M algorithm, omnibus association test, and minor haplotype frequency of 0.01. No adjustment was made for age or sex.

PLINK estimated haplotype frequencies for all possible haplotypes in each window through statistical inference, employing the expectation-maximization likelihood algorithm [59,60]. This method was used to estimate maximum likelihood when data were incomplete or hidden. In our case, the hidden data were the actual haplotypes, which could not be identified since although genotypes were known, the chromosome-phasing was not. The *p*-value criteria generally used for statistical significance in GWAS has been *p* < 5 × 10^−8^ [61]. However, since in haplotype analysis, the same SNP was tested in all possible haplotypes involved using 1–25 SNPs at a time, where the SNP would occur first, second, third, etc., as the window slides, it meant that all those tests concern the same locus and were not considered independent tests, which needed to be corrected for. It also meant that, theoretically, since all SNPs were chosen to have at least two alleles, the maximum possible number of haplotypes including the same SNP (locus) was 2^50^. The number of possible haplotypes (based on the sample set of cases and controls) was specified for each window and rarely exceeded 50. Therefore, a *p*-value criterion for statistical significance of *p* < 2.5 × 10^−6^ was used for this study. Each SNP was tested multiple times for each value of the specific SNP. Thus, if all SNPs had two possible genotypes, in total, 2^50^ possible haplotypes were generated, analyzing first only one SNP, then two SNPs, then three, and up to 25 SNPs. This approach generated several haplotypes representing the same unique haplotype varying in lengths from 1 to 25 SNPs. This meant that, depending on the variability of SNPs within these different haplotypes, the number of haplotypes generated for each SNP varied. For example, the first significant locus at chromosome 1 is described in Table 3 below (Supplementary Table S1). There were several haplotypes of varying lengths, which could be defined as parts of three unique haplotypes (Table 3). Only one fulfilled our criterion for statistical significance (in box); this one and what likely were parts of the same haplotype are all in bold, a second haplotype marked with *, and a third haplotype marked with ** (Table 3). 

## Figures and Tables

**Table 1:** Haplotypes with *p* < 2.5 × 10^−6^ found in the CoGa Study.

CHR	BP1	BP2	HF	OR	Stat	*p*-Value	Genes
1p34.2	40258993	40513710	0.02	3.54	23.5	1.28 × 10^−6^	*TRIT1*, *MYCL*, *MFSD2A*, *CAP1*
1q23.3	161976234	162160920	0.01	4.39	22.3	2.36 × 10^−6^	*OLFML2B*, *NOS1AP*
1q25.1	175135829	175339021	0.01	4.41	27.9	1.30 × 10^−7^	*KIAA0040*, *TNR*
2p25.3	2956085	3108215	0.02	3.96	24.9	6.01 × 10^−7^	
2p21	42494273	42708267	0.05	2.22	23.3	1.36 × 10^−6^	*EML4*, *COX7A2L*, *KCNG3*
2p12	77360561	77670072	0.01	4.87	24.7	6.54 × 10^−7^	*LRRTM4*
2p12	78945755	79096243	0.02	3.84	23.7	1.13 × 10^−6^	
2q33.1	202688907	202839768	0.02	4.03	26.1	3.28 × 10^−7^	* *CDK15*
2q36.1	222785645	222877384	0.06	2.13	24.5	7.58 × 10^−7^	
2q36.2	225848579	226023294	0.05	2.10	23.5	1.22 × 10^−6^	* *DOCK10*
2q37.3	241879702	241943575	0.05	2.16	22.5	2.09 × 10^−6^	* *CROCC2*, *SNED1*
3p24.1	30547439	30625123	0.04	2.32	22.2	2.46 × 10^−6^	
3p22.2	38737643	38830893	0.02	3.23	22.7	1.91 × 10^−6^	*SCN10A*
4q12	54556087	54657790	0.01	5.14	22.2	2.45 × 10^−6^	*LNX1*
4q13.1	63250043	63345570	0.02	2.98	25.1	5.33 × 10^−7^	
4q28.3	137657271	137808928	0.01	4.28	26.6	2.47 × 10^−7^	
4q31.3	154605745	154807201	0.01	3.97	24.1	9.03 × 10^−7^	* *TLR2*, *RNF175*, *SFRP2*
4q34.3	183027022	183081780	0.03	2.93	22.8	1.84 × 10^−6^	*TENM3*
4q35.1	183938119	184215675	0.01	5.98	24.2	8.62 × 10^−7^	* *WWC2*
5q13.1	67317114	67430447	0.08	1.88	22.3	2.34 × 10^−6^	
5q21.2	103143532	103253764	0.03	2.95	23.5	1.25 × 10^−6^	
5q35.2	173970989	174050806	0.01	5.08	24.2	8.69 × 10^−7^	
5q35.2	174768731	174836280	0.01	4.66	23.6	1.17 × 10^−6^	
6p25.1	5159064	5265853	0.12	1.75	23.0	1.62 × 10^−6^	*LYRM4*, *FARS2*
6p21.1	45705079	45795743	0.02	3.64	24.8	6.35 × 10^−7^	*
7p21.3	9146717	9235662	0.03	3.18	22.5	2.14 × 10^−6^	
7p15.1	28891257	28903516	0.03	3.23	24.9	6.06 × 10^−7^	
7q11.22	70082913	70137165	0.02	3.26	24.4	7.69 × 10^−7^	*AUTS2*
7q11.22	71279081	71482823	0.04	2.24	22.4	2.19 × 10^−6^	*CALN1*
7q21.13	89107084	89377947	0.03	3.46	28.5	9.35 × 10^−8^	
7q22.1	103162847	103222306	0.02	4.51	29.2	6.55 × 10^−8^	* *RELN*
7q31.31	120073573	120408252	0.01	4.79	27.4	1.69 × 10^−7^	*KCND2*
7q36.3	158726655	158845384	0.11	1.83	23.3	1.41 × 10^−6^	*DYNC2I1*, *VIPR2*
8q24.3	140876251	140924076	0.03	2.77	22.2	2.43 × 10^−6^	*TRAPPC9*
9q22.31	95772228	95854695	0.33	1.48	22.2	2.42 × 10^−6^	*FGD3*, *SUSD3*
9q34.3	137762857	137850460	0.01	4.95	29.4	5.90 × 10^−8^	* *FCN2*, *FCN1*
10q22.3	80462677	80491731	0.02	3.11	23.4	1.30 × 10^−6^	
10q25.3	115049710	115119640	0.01	4.34	23.6	1.21 × 10^−6^	
11q23.3	117525420	117653955	0.02	3.50	22.2	2.49 × 10^−6^	*DSCAML1*
11q23.3	117690400	117722742	0.03	2.58	25.1	5.42 × 10^−7^	*FXYD2*, *FXYD6*-*FXYD2*, *FXYD6*
12p13.32	4384696	4397766	0.02	4.05	22.5	2.05 × 10^−6^	*CCND2*
12q24.21	115432175	115453535	0.05	2.60	27.4	1.67 × 10^−7^	
12q24.33	129548211	129631493	0.02	3.31	22.5	2.11 × 10^−6^	*TMEM132D*
13q12.11	20407151	20686272	0.02	2.81	23.4	1.29 × 10^−6^	* *ZMYM5*, *ZMYM2*
13q22.1	74316318	74347673	0.07	1.91	22.3	2.29 × 10^−6^	* *KLF12*
14q24.1	67989097	68056390	0.01	4.26	26.4	2.74 × 10^−7^	*TMEM229B*, *PLEKHH1*, *PIGH*
14q24.2	73458661	73471257	0.15	1.71	22.5	2.07 × 10^−6^	*ZFYVE1*
15q11.2	25037506	25102392	0.04	2.66	26.1	3.28 × 10^−7^	*SNRPN*
15q25.1	81357747	81454021	0.04	2.37	25.9	3.65 × 10^−7^	*CFAP161*, *IL16*
17p13.3	2797013	2885146	0.01	7.39	23.9	1.03 × 10^−6^	*RAP1GAP2*
17p13.1	8243661	8558728	0.01	4.50	23.9	9.90 × 10^−7^	*ODF4*, *KRBA2*, *RPL26*, *RNF222*, *NDEL1*, *MYH10*
17p12	14317939	14362003	0.05	2.33	27.9	1.27 × 10^−7^	
17q21.32	46348384	46355550	0.02	3.10	22.7	1.86 × 10^−6^	* *SKAP1*
17q22	54427831	54749558	0.02	3.93	22.9	1.72 × 10^−6^	*ANKFN1*, *NOG*
18p11.31	3344851	3471640	0.01	5.43	22.8	1.84 × 10^−6^	*TGIF1*
18q22.2	67322660	67432141	0.14	1.68	22.4	2.20 × 10^−6^	*DOK6*
20q13.31	54984064	55109304	0.03	2.59	22.5	2.14 × 10^−6^	*CASS4*, *RTF2*, *GCNT7*, *FAM209A*, *FAM209B*
20q13.33	60926223	60927349	0.67	1.55	24.1	9.36 × 10^−7^	*LAMA5*
20q13.33	60929539	60930093	0.67	1.53	23.1	1.55 × 10^−6^	*LAMA5*
20q13.33	60938228	60938310	0.67	1.53	22.5	2.08 × 10^−6^	*LAMA5*
20q13.33	60964857	60966686	0.65	1.52	24.0	9.47 × 10^−7^	* *CABLES2*
20q13.33	60970675	60973432	0.65	1.50	22.6	2.03 × 10^−6^	* *CABLES2*
20q13.33	61628970	61646108	0.02	3.29	23.4	1.30 × 10^−6^	*BHLHE23*
21q21.2	24274393	24462002	0.03	2.82	26.5	2.60 × 10^−7^	* *MIR6130*
22q13.2	43596125	43629089	0.01	4.57	22.3	2.39 × 10^−6^	*SCUBE1*

CHR—Chromosome and karyotype band. BP1 and BP2—First and last position in the haplotype (GRCh37). HF—Haplotype frequency in sample. OR—Odds ratio. Stat—Z value from Wald test. * Haplotype previously identified in the CoGaPro2 study.

**Table 2:** Demographics of the CRC cases in the GWAS.

**CRC Cases with Gastric Cancer in Their Families**	
Family:	83, 21, and 322 cases with one, two, or no relatives with CRC
Sex:	213 women and 213 men
Age:	13 early-onset (<50), 413 late-onset (≥50)
Location:	47 caecum, 242 left colon, 137 right colon
Stage:	60 Dukes A, 138 Dukes B, 104 Dukes C, and 124 Dukes D
**CRC Cases with Prostate Cancer in their Families**	
Family:	62, 26, and 236 cases with one, two, or no relatives with CRC
Sex:	142 women and 182 men
Age:	23 early-onset, 301 late-onset
Location:	43 caecum, 201 left colon, 50 right colon, 19 unknown
Stage:	43 Dukes A, 96 Dukes B, 87 Dukes C, 35 Dukes D, and 53 unknowns

**Table 3:** Haplotypes for the first significant haplotype in the results.

Hap	BP1	BP2	Haplotype	F	OR	Stat	*p*
6	40258993	40288051	**AGA**	0.30	1.18	3.99	4.58 × 10^−2^
8	40258993	40290277	**AGAG**	0.29	1.18	4.15	4.16 × 10^−2^
10	40258993	40302463	**AGAGC**	0.28	1.20	4.71	2.99 × 10^−2^
11	40258993	40303627	**AGAGCG**	0.28	1.20	4.95	2.61 × 10^−2^
12	40258993	40306898	**AGAGCGA**	0.28	1.22	5.49	1.91 × 10^−2^
14	40258993	40316155	**AGAGCGAG**	0.28	1.22	5.72	1.68 × 10^−2^
14	40258993	40324210	**AGAGCGAGA**	0.28	1.22	5.70	1.70 × 10^−2^
14	40258993	40362066	**AGAGCGAGAC**	0.28	1.22	5.85	1.55 × 10^−2^
15	40258993	40364803	**AGAGCGAGACC**	0.27	1.20	4.82	2.82 × 10^−2^
15	40258993	40383552	**AGAGCGAGACCC**	0.26	1.23	6.35	1.17 × 10^−2^
18	40258993	40389420	**AGAGCGAGACCCG**	0.06	1.45	5.36	2.07 × 10^−2^
19	40258993	40395169	**AGAGCGAGACCCGA**	0.04	1.59	6.10	1.35 × 10^−2^
24	40258993	40433771	**AGAGCGAGACCCGAG**	0.02	2.27	6.16	1.31 × 10^−2^
24	40258993	40433771	GAGGCGAGACCCGAG *	0.05	1.45	4.05	4.42 × 10^−2^
24	40258993	40435999	**AGAGCGAGACCCGAGG**	0.01	2.31	5.50	1.90 × 10^−2^
24	40258993	40435999	GAGGCGAGACCCGAGG *	0.05	1.50	4.87	2.73 × 10^−2^
22	40258993	40437923	**AGAGCGAGACCCAAGGA**	0.14	1.35	8.20	4.20 × 10^−3^
22	40258993	40437923	GAGACGAGACCCAAAGG **	0.03	0.57	3.89	4.86 × 10^−2^
21	40258993	40458920	**AGAGCGAGACCCAAGGAA**	0.13	1.43	11.30	8.00 × 10^−4^
19	40258993	40499302	**AGAGCGAGACCCAAGGAAA**	0.02	2.60	15.80	7.00 × 10^−5^
19	40258993	40504550	**AGAGCGAGACCCAAGGAAAA**	0.02	3.23	20.40	6.00 × 10^−6^
19	40258993	40513710	**AGAGCGAGACCCAAGGAAAAA**	0.02	3.54	23.50	1.00 × 10^−6^
20	40258993	40543019	**AGAGCGAGACCCAAGGAAAAAA**	0.02	3.39	21.20	4.00 × 10^−6^
19	40258993	40547950	**AGAGCGAGACCCAAGGAAAAAAG**	0.02	3.46	21.90	3.00 × 10^−6^
19	40258993	40547950	GAGACGAGACCCAAAGGAGAGAA **	0.02	0.46	4.30	3.81 × 10^−2^

From Supplementary Table S1. There were three possible unique haplotypes. One unique haplotype, all variants in bold, only one statistically significant in box, a second unique haplotype marked with *, and a third unique haplotype marked with **; Hap—number of unique haplotypes for the window; BP1, BP2—base pair of first and last SNP in the haplotype; F—estimated frequency of the number of haplotypes in the sample (cases and controls); OR—odds ratio; Stat—Z value from Wald test; *p*—*p*-value.

## Data Availability

Access to the data is controlled. Variants that fulfill our selection criteria can be found in the Supplemental Tables. However, Swedish laws and regulations prohibit the release of individual and personally identifying data. Therefore, the whole dataset cannot be made publicly available. The data that support the findings of this study are available from the corresponding authors upon a reasonable request.

## Supplementary Materials

Supporting Information can be downloaded at: https://zenodo.org/records/18054099 (accessed on 26 December 2025).

## Abbreviations

The following abbreviations are used in this manuscript:

BP	Base pair
CHR	Chromosome
CI	Confidence interval
CNS	Central nervous system
CRC	Colorectal cancer
DNA	Deoxyribonucleic acid
FAP	Familial adenomatous polyposis
GWAS	Genome-wide association study
HF	Haplotype frequency
MSI	Microsatellite instability
OR	Odds ratio
PSA	Prostate-specific antigen
SNP	Single-nucleotide polymorphism
